# Polymerase acidic subunit of H9N2 polymerase complex induces cell apoptosis by binding to PDCD 7 in A549 cells

**DOI:** 10.1186/s12985-021-01547-7

**Published:** 2021-04-13

**Authors:** Shaohua Wang, Na Li, Shugang Jin, Ruihua Zhang, Tong Xu

**Affiliations:** Key Laboratory of Preventive Veterinary Medicine, Department of Veterinary Medicine, Animal Science College, Hebei North University, Zhangjiakou, 075131 Hebei People’s Republic of China

**Keywords:** H9N2, Influenza A virus, PA subunit, Programmed cell death protein 7, Cell apoptosis

## Abstract

**Background:**

H9N2 influenza virus, a subtype of influenza A virus, can spread across different species and induce the respiratory infectious disease in humans, leading to a severe public health risk and a huge economic loss to poultry production. Increasing studies have shown that polymerase acidic (PA) subunit of RNA polymerase in ribonucleoproteins complex of H9N2 virus involves in crossing the host species barriers, the replication and airborne transmission of H9N2 virus.

**Methods:**

Here, to further investigate the role of PA subunit during the infection of H9N2 influenza virus, we employed mass spectrometry (MS) to search the potential binding proteins of PA subunit of H9N2 virus. Our MS results showed that programmed cell death protein 7 (PDCD7) is a binding target of PA subunit. Co-immunoprecipitation and pull-down assays further confirmed the interaction between PDCD7 and PA subunit. Overexpression of PA subunit in A549 lung cells greatly increased the levels of PDCD7 in the nuclear and induced cell death assayed by MTT assay.

**Results:**

Flow cytometry analysis and Western blot results showed that PA subunit overexpression significantly increased the expression of pro-apoptotic protein, bax and caspase 3, and induced cell apoptosis. However, knockout of PDCD7 effectively attenuated the effects of PA overexpression in cell apoptosis.

**Conclusions:**

In conclusion, the PA subunit of H9N2 virus bind with PDCD7 and regulated cell apoptosis, which provide new insights in the role of PA subunit during H9N2 influenza virus infection.

**Supplementary Information:**

The online version contains supplementary material available at 10.1186/s12985-021-01547-7.

## Introduction

Influenza is one of the most common infectious diseases, which mainly caused by the infection of influenza virus in seasonal epidemics [1, 2]. There are four types of influenza viruses (A to D), belonging to the RNA virus family Orthomyxoviridae [3, 4]. Avian influenza A virus mainly infects poultry, and some subtypes also infect mammals and humans. Different subtypes of influenza A virus come from different combinations of surface proteins, haemagglutinin (HA) and neuraminidase (NA) [3]. Among them, a subtype of influenza virus of influenza A, such as H9N2, induces avian influenza, which is a form of respiratory infectious disease [5, 6]. The H9N2 subtype avian influenza virus has been reported to be low-pathogenic. Infection of H9N2 virus displays no clinical illness or only mild defects, such as a slight drop in egg production or mild respiratory signs, unless the infection is mixed with other pathogens, which will lead to severe respiratory syndromes and a high mortality rate [7]. Therefore, H9N2 avian influenza is a severe public health risk and causes a huge economic loss to poultry production.

It is known that the genome of influenza A virus consists of eight ribonucleoproteins (RNPs), in which includes the genomic RNA, nucleoprotein, and an RNA polymerase [8]. The RNA polymerase in influenza A virus is a heterotrimer that composes of polymerase acidic (PA), polymerase basic 1 (PB1) and PB2. Accumulating studies have revealed that the polymerase complex is necessary for the viral host selection, replication, pathogenicity, and transmission [9–14]. Moreover, PA subunit of the RNA polymerase was reported to contribute to overcome the host species barrier and adapt to the new host species [15]. In addition, the polymorphisms in PA protein has also been identified to affect the pathogenicity and host adaptation of influenza A virus [16]. The PA subunit of the RNA polymerase is an endonuclease and a protease that induces proteolytic degradation of co-expressed proteins [17, 18]. PA Subunit was reported to interact with a cellular protein with homology to a family of transcriptional activators [19]. However, other bind targets of PA subunit still need to be elucidated.

In the present study, mass spectrometry was employed to screen for the binding protein with PA subunit of viral RNA polymerase. We identified that programmed cell death protein 7 (PDCD7) is a new binding target of PA subunit of H9N2 virus. Gain- and loss-of-function experiments demonstrated that PA subunit interacting with PDCD7 can activate apoptosis pathway and induce cell apoptosis, which may provide new insights in the role of PA subunit during H9N2 influenza virus infection.

## Material and methods

### Cells and cell culture

A549 cells were purchased form American Type Culture Collection (ATCC® CCL-185™). Cells were cultured with McCoy’s 5A Media (Modified with Tricine) supplemented with 10% fetal bovine serum (FBS) and 1% penicillin–streptomycin solution in a 37 °C incubator with an atmosphere of 5% CO_2_ and 95% air. Cells were passaged by trypsinization.

### Mass spectrometry

For mass spectrometry analysis, lysates from A549 cells transfected with Flag-tagged PA or vector control cells were prepared and incubated with Flag-M2 agarose beads (Sigma) overnight at 4 °C. The beads were then washed 3 times with IP wash buffer (150 mM NaCl, 10 mM HEPES pH 7.4, 0.1% NP-40), followed by elution with 1 M glycine (pH 3.0) twice. The eluted proteins were denatured and separated on SDS–polyacrylamide gels and stained with Coomassie blue; the indicated bands were subjected to mass spectrometry analysis.

### Co-immunoprecipitation

Firstly, PA subunit of H9N2 polymerase were overexpressed in A549 cells and cultured for 48 h, and then were collected via trypsinization. Total cell lysates were made with NP-40 lysis buffer and was quantitated by bicinchoninic acid (BCA). The overexpressed protein was precipitated by adding Flag-M2 beads and incubating at 4 °C overnight, then was isolated via centrifugation. The immunoprecipitation samples were analysed by SDS-PAGE and western blot.

### Pull-down assays

Total protein lysates from A549 cells were as aforementioned. The purified GST-tagged protein was added into the cell lysates to bind interacted partners at 4 °C, GST beads were added to enrich the tagged complex, and the unspecific binding proteins were removed by multiple wash with washing buffer containing Tris–HCl and detergent. The samples were analysed by SDS-PAGE and western blot.

### Cell Viability assay

A549 cells were plated in 96-wells plated with complete medium with FBS and antibiotics, and the proliferation was analysed by Cell Counting Kit-8 (CCK-8). Briefly, 20 μl CCK-8 reagent was added into each well and incubated at 37 °C for 1 h, absorbance at 450 nM was then collected by the Molecular Device M2. Data was analyzed by GraphPad Prism 6.0.

### Flow cytometry analysis

Cell apoptosis was analyzed by Annexin V and PI staining. Briefly, A549 cells were trypsinized and washed with PBS. Annexin V and PI reagent were added and incubated for 30 min form light. The fluorescence signals were analysed by BD Accuri™ C6.

### Quantitative real-time PCR.

The total RNA of A549 cells was extracted by Trizol method. Briefly: add 1 mL Trizol to the cell culture dish to lyse and collect the cells, then add chloroform at the ratio of 200 μL/1 mL Trizol, shake vigorously for 15 s, stand for 5 min and then centrifuge; take the upper aqueous phase into a new centrifuge tube and add equal volume of isopropanol to precipitate the RNA. The precipitate was collected by centrifugation after standing for 30 min at 4 °C. The precipitate was washed with 75% ethanol without RNase and DNase and centrifuged again. The supernatant was discarded and dried and then dissolved in ultrapure water without RNase and DNase. cDNA was synthesized by reverse transcription form 2 μg of RNA, and 1 μL of the synthesized cDNA was used as the template for real-time fluorescence quantitative PCR detection. PCR primers were as followed: GAPDH sense 5′- CAA GTT CAA CGG CAC AGT CA-3′, GAPDH antisense 5′- ATC TCG CTC CTG GAA GAT GG-3′; PDCD7 sense primer: 5′- GGG ATC CAG ATG AGT TCC CA-3′, PDCD7 antisense primer: 5′- ACG AAG TTG CCT TTG GGA TG-3’.

### Western blot

Proteins samples were firstly separated with SDS-PAGE and were transferred onto PVDF membranes. Sequentially, the membranes were blocked with 5% non-fat milk and then were incubated with Primary antibodies overnight at 4 °C, Horseradish Peroxidase (HRP) conjugated secondary antibody was added and incubated for 1 h at room temperature. Chemiluminescence signalling was collected via adding ECL reagents and the images was processed and analyzed with photoshop.

### CRISPR/Cas9 gene editing technology

Guide RNA targeting the PDCD7 coding sequence was design on websites (Benchling.com) and was synthesized by micro-helix. PX459 vector containing both gRNA backbone and spCas9 coding sequence was used to clone the designed gRNA and was confirmed via sanger sequencing. The gene editing plasmid was transfected into A549 cells using Lipo3000 and then were selected with puromycin at 1ug/ml for 3 days. The survived cells were plated at low concertation allowing them into single clones after 2 weeks culture, and the knockout alleles were confirmed by Sanger sequencing and western blot.

### Virus preparation

Isolation of the A/Swine/HeBei/012/2008/(H9N2) virus was previously described [20]. For virus propagation, chicken embryos of 10-day-old which were free of pathogens were infected (Beijing Laboratory Animal Research Center, Beijing, China) and passaged for about 3 generations. The virus were then isolated by centrifugation and store at 80 °C.

### Statistical analysis

All the data were expressed as mean ± standard deviation. T test and a one-way ANOVA was used for the statistical analysis was performed by GraphPad 6.0.

## Results

### PDCD7 is a binding target of PA subunit of H9N2 virus

**Fig. 1:**
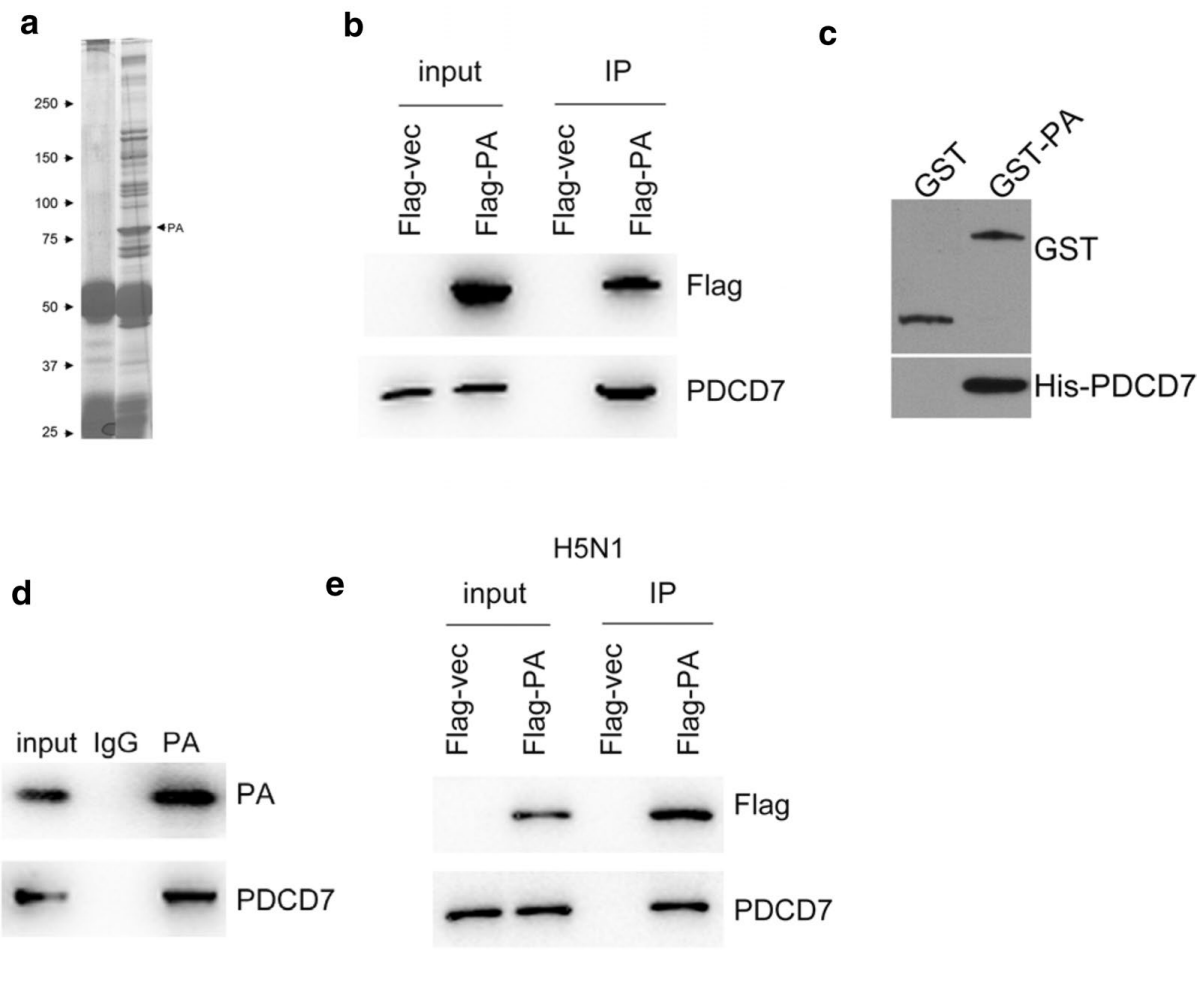
PDCD7 is a binding target of PA subunit of H9N2 virus. **a** Immunoprecipitation fractions were separated by SDS-PAGE and stained with Coomassie blue; the exogenous expressed Flag-PA was indicated by arrow. **b** Western blot detection of PDCD7 in the immunoprecipitation fraction of exogenous expressed Flag-PA; Flag-M2 beads were used to isolate Flag-tagged PA subunit and the Flag-vector was used as negative control. **c** Western blot detection of His-tagged PDCD7 in the fraction of GST-PA pull-down. The GST protein was used as negative control. **d** Western blot result showed the endogenous interaction between PDCD7 and PA subunit of the H9N2 virus in A549 cells. The polyclonal antibody for PA was purified from mouse. E. Western blot result shown interaction between PDCD7 and the PA subunit from H5N1. The Flag-vector was used as a negative control

To investigate the role of the RNA polymerase in the RNP complex during the infection of H9N2 influenza virus, mass spectrometry (MS) was performed to search the binding protein of the RNA polymerase (Fig. 1a). We found that programmed cell death protein 7 (PDCD7) was pulled down by PA subunit antibody and identified by MS, but other isoforms of PDCD were not observed, suggesting that PDCD7 may be a potential binding target of PA subunit.

Next, co-immunoprecipitation assay was performed to examine the interaction between PDCD7 and PA subunit. First, PDCD7 was co-transfected with PA-Flag tag or empty vector into A549 cells. The immunoprecipitation was performed by using anti-Flag antibody and then blotted with PDCD7 antibody. Interestingly, PDCD7 signal was observed in the PA-Flag immunoprecipitates but not in vector control group (Fig. 1b), demonstrating an interaction between PDCD7 and PA subunit. To check whether this interaction is direct, we first purified PA with GST tag in *E.coli* cells and attempted to pull down His-tagged PDCD7 by using anti-His antibody. As shown in Fig. 1c, recombinant GST-PA can pulldown His-PDCD7, confirming that PDCD7 is a direct binding target of PA subunit of H9N2 virus. To verify if this interaction happens under pathological conditions, we used in-house produced PA poly antibodies for the immunoprecipitation assay, and the presence for PDCD7 was observed in the PA-fraction as shown in Fig. 1d. Considering the conservation of PA subunits among different viruses, we nest checked the interaction between PDCD7 and the PA subunit from H5N1 virus, and the interaction was observed as shown in Fig. 1e. Collectively, interaction between PDCD7 and the PA subunit from influenza virus might be a universal mechanism.

### PA subunit of H9N2 virus greatly elevates the level of PDCD7 and induces cell death in lung cells

**Fig. 2:**
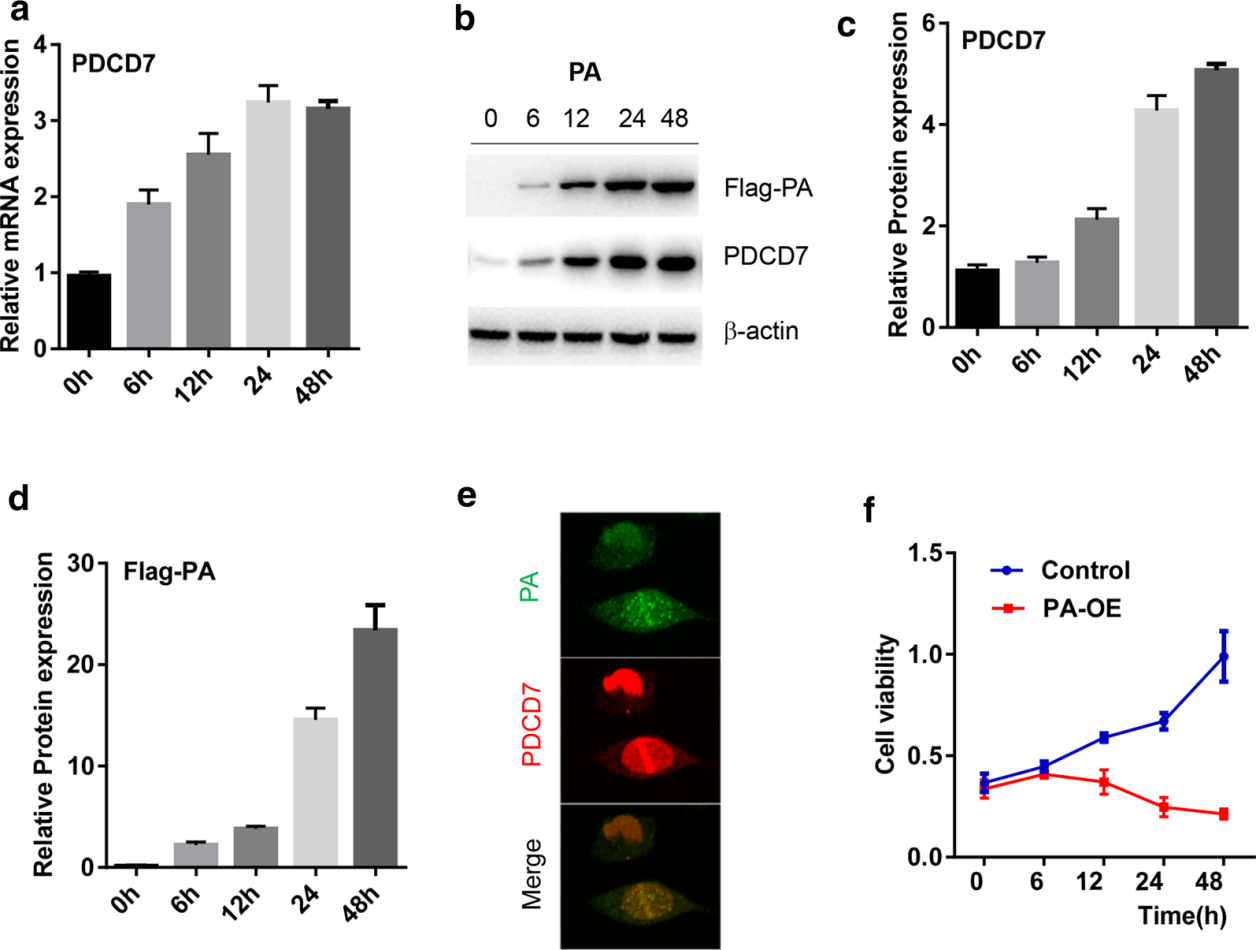
PA subunit of H9N2 virus greatly elevates the level of PDCD7 and induces cell death in lung cells. **a** Quantitative analysis of PDCD7 mRNA expression in lung cells upon PA overexpression in A549 cells. B-D. Western blot analysis of PDCD7 expression in A549 cells upon Flag-PA overexpression (**b**); the relative expression of PDCD7 (**c**) and Flag-PA (**d**) were quantitated by Image J. **e** Immunofluorescence demonstrated colocalization of GFP-PA and RFP-PDCD7 in A549 cells. **f** Cell viability of A549 cells upon PA overexpression by CCK-8, PA-OE: PA overexpression

To examine whether overexpress PA subunit of H9N2 virus affects the expression level of PDCD7, the expression vector containing PA with Flag tag was transfected into A549 lung cells for 6 h, 12 h, 24 h and 48 h. As shown in Fig. 2A, quantitative real-time PCR revealed that the expression of PDCD7 began to upregulate at 6 h and reached to a peak at 24 h. Western blot results showed similar expression pattern of PDCD7 after PA overexpression (Fig. 2b–d), suggesting that overexpress PA subunit of H9N2 virus significantly upregulated PDCD7 expression in a time-dependent manner. Furthermore, immunohistochemistry results showed that PDCD7 signals (GFP) colocalized with PA signals (RFP) in the nuclear (Fig. 2e), although PA signals also existed in the plasma that may have other functions. These results indicated that PA subunit of H9N2 virus not only bind with PDCD7, but also elevated the expression level of PDCD7 in the nuclear.

Next, MTT assay was performed to determine the cell viability in A549 lung cells after overexpress PA subunit of H9N2 virus. Consistent with the change of PDCD7 expression, the cell viability began to drop at 12 h and reached to a peak at 24 h (Fig. 2f), suggesting that PA overexpression induced cell death through binding and upregulating PDCD7.

### Overexpression of PA subunit induced cell apoptosis

**Fig. 3:**
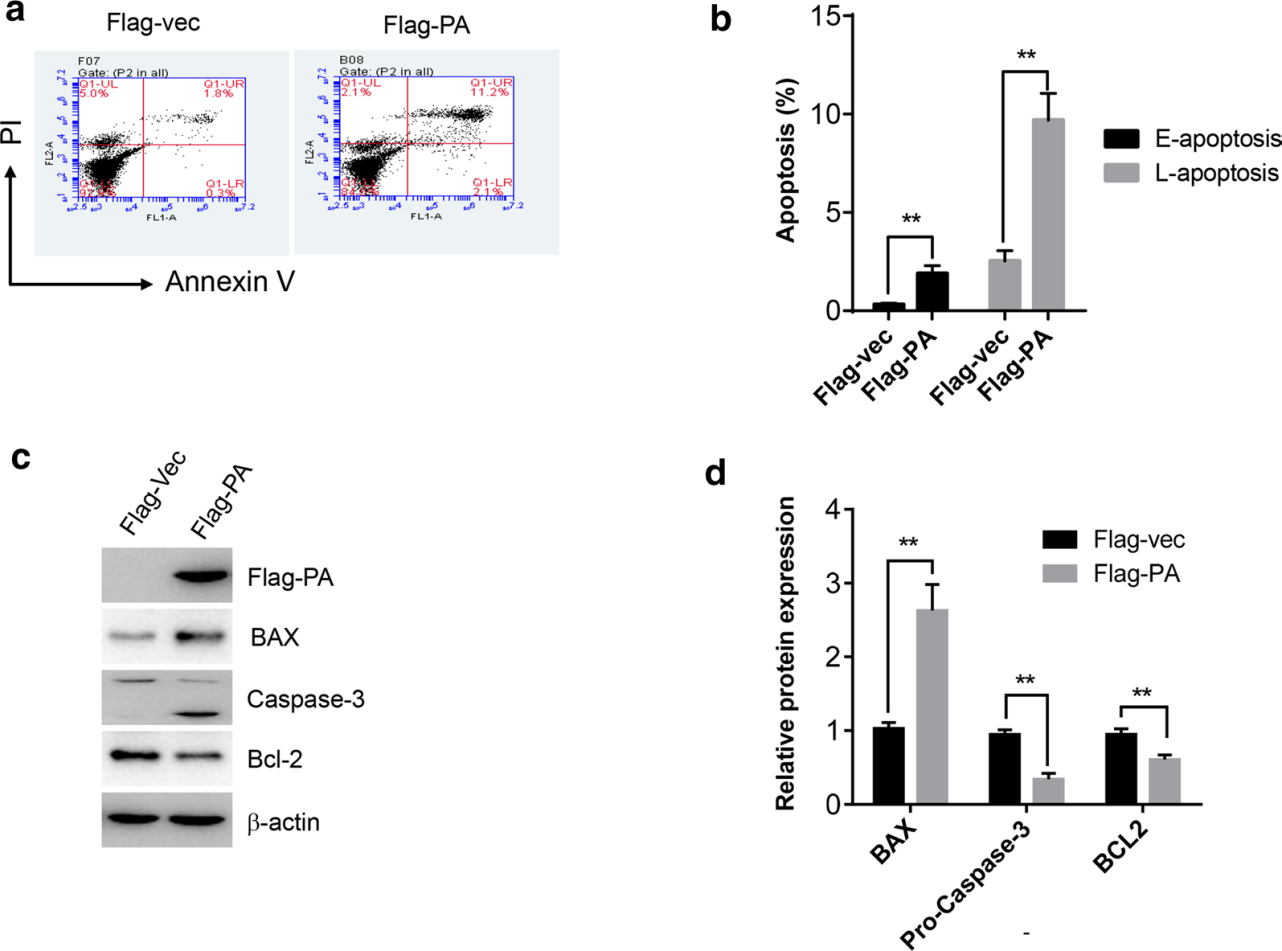
Overexpression of PA subunit induced cell apoptosis. **a**–**b** Flow cytometry analysis of cell apoptosis by Annexin V and PI staining in A549 cells overexpressed with Flag-PA, Early apoptosis: E-apoptosis; Late apoptosis: L-apoptosis. **c** Western blot analysis of apoptosis related proteins in A549 cells. **d** quantitative results of apoptotic related proteins by Image J. “**” means P < 0.01

PDCD7 is a protein that mediates cell apoptosis in response to various stimuli. In addition, H9N2 infection leads to severe apoptosis in A549 cells as shown in Additional file 1: Fig. 1a, b. To understand whether PA-overexpression-induced cell death resulted from cell apoptosis, annexin-PI staining combining with flow cytometry analysis was performed to determine the cell apoptosis. As shown in Fig. 3a, b, overexpression of PA subunit significantly induced cell apoptosis. Moreover, Western blot analysis demonstrated that the expression level of pro-apoptotic proteins, Bax and cleaved caspase-3, was significantly increased after PA overexpression, while the expression level of anti-apoptotic protein, Bcl-2, was decreased, compared to the vector control group (Fig. 3c, d), suggesting that overexpression of PA subunit activated apoptotic pathway and induced cell apoptosis.

### PDCD7 mediates the PA-overexpression-induced cell apoptosis in Lung A549 cells

**Fig. 4:**
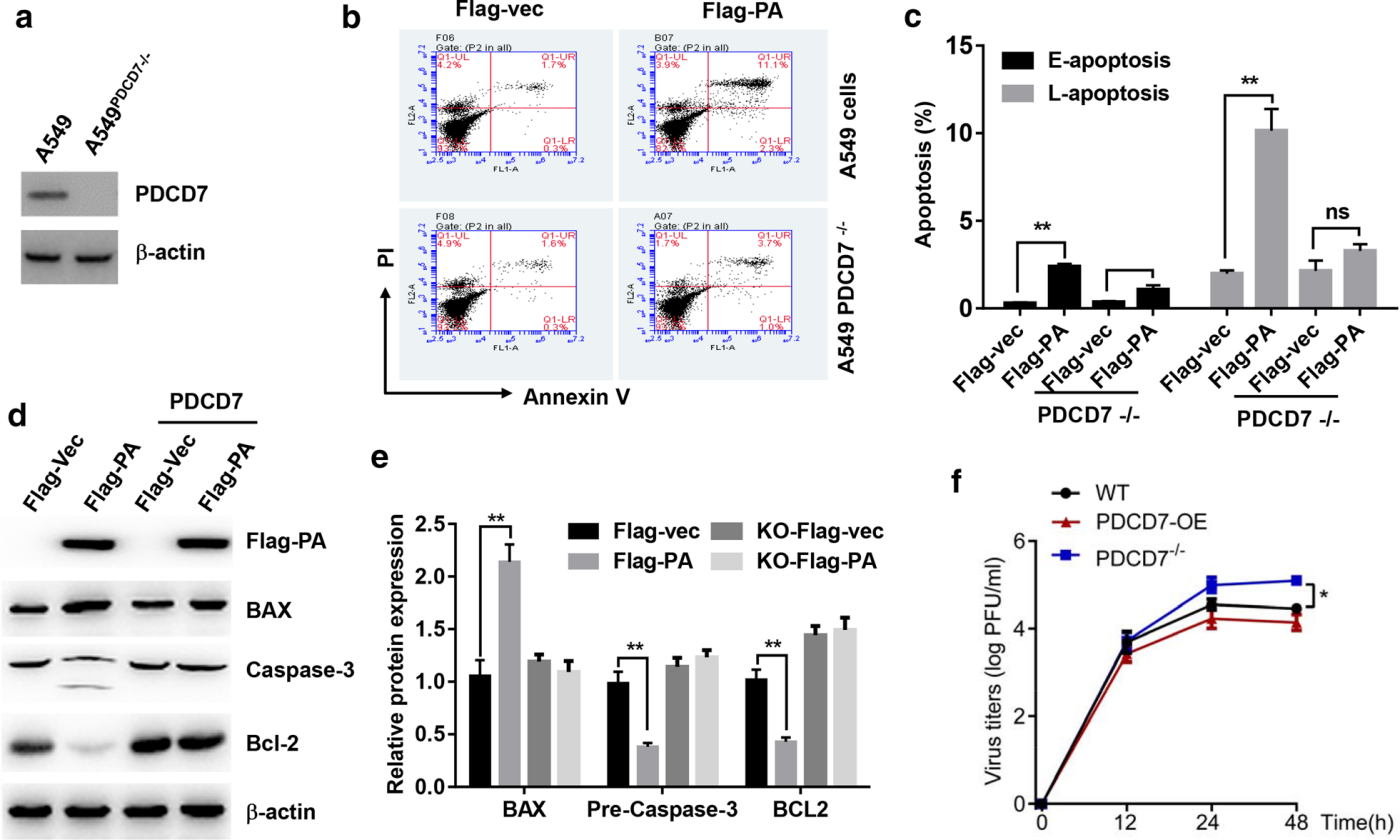
PDCD7 mediates the PA-overexpression-induced cell apoptosis in Lung A549 cells. **a** Western blot analysis of PDCD7 knockout A549 cells, the b-actin was used as internal control. **b** Flow cytometry analysis of apoptosis in wide type A549 cells or PDCD7 knockout cells upon overexpression with Flag-vector of Flag-PA. **c** Quantitative results of cells at different stages. Early apoptosis: E-apoptosis; Late apoptosis: L-apoptosis. **d** Western blot analysis of apoptosis related protein expression in A549 cells or PDCD7 knockout cells upon overexpression with Flag-vector of Flag-PA. **e** quantitative results of apoptotic related proteins by Image J. “**” means P < 0.01. **f** Titers of the infected H9N2 virus in wide type A549 cells or PDCD7 knockout A549 cells, “*” means P < 0.05

To investigate whether PDCD7 mediated PA-overexpression-induced cell apoptosis in Lung A549 cells, CRISPR/Cas9 gene editing technology was used to selectively knock out PDCD7 expression. Western blot results showed that PDCD7 expression were successfully knocked out in Lung A549 cells (Fig. 4a). Next, PA-Flag tag vector was transfected into the PDCD7 knockout cell lines for 24 h. Immunohistochemistry assay showed that PA signal was still observed in the nuclear, but do not bind with PDCD7 (Fig. 4b). Interestingly, PA-overexpression-induced cell apoptosis was great attenuated in PDCD7 knockout cell lines (Fig. 4b, c). Moreover, PA-overexpression-induced upregulation of BAX, cleaved caspase-3, and downregulation of Bcl-2 were also dramatically diminished (Fig. 4d, e). These results indicated that PDCD7 was downstream signal of PA subunit of H9N2 virus to activate cell apoptosis pathway and induce cell apoptosis. To check if the PDCD7 affects the replication of H9N2 virus in A549 cells, we then evaluated the titers of H9N2 virus in both cell lines. The replication of H9N2 was slightly increased in PDCD7 knockout A549 cells, as demonstrated by higher titer in the PDCD7 absent cells (Fig. 4f). However, overexpression of PDCD7 in A549 cells did not significantly suppressed the replication of the H9N2 virus.

## Discussion

The avian H9N2 influenza virus belongs to the influenza A virus and was first isolated from the turkeys in Wisconsin in 1966 [21, 22]. Since then, H9N2 virus becomes one of the most prevalent avian influenza viruses, causing repeated poultry and human infection [2, 7, 23]. Although the H9N2 influenza virus has diversified into multiple lineages, H9N2 virus was reported to be low-pathogenic unless the viral infection is complicated with other pathogens [7, 24]. Avian H9N2 viruses can overcome the host species barrier and infect other mammals and humans [15, 25, 26]. Accumulating studies have revealed that PA subunit of the RNA polymerase in influenza A virus are necessary in overcoming the host species barrier and host adaptation [15–19, 27–31], indicating that PA subunit is critical for the infection of avian H9N2 influenza virus. In the present study, combining MS with pull-down assay, we identified that PA subunit is capable of nuclear transport and binding with PDCD7 to activate apoptotic pathway in A549 lung cells.

The PA subunit of the RNA polymerase in influenza A virus is an endonuclease and a protease that induces proteolytic degradation of co-expressed proteins [17, 18, 26]. It has been reported that amino acid residues in the N-terminus of the PA subunit is critical for the protein stability, endonuclease activity, cap binding, and protein binding [22, 32]. Mutations in the PA subunit has been found to inhibit endonucleolytic cleavage of capped RNAs [33, 34]. Moreover, PA subunit of the RNA polymerase was also revealed to contribute to overcome the host species barrier and adapt to the new host species [15]. Mutations of PA subunit, such as PA-K356R, are necessary for viral adaptation to new hosts, and might render H9N2 viruses adapted for human infection [35]. In addition, the polymorphisms in PA protein also affects the pathogenicity and host adaptation of influenza A virus [16]. These evidences indicated that the PA subunit are critical for the function and infection of influenza A virus. A few binding proteins were identified to bind with PA subunit, such as a cellular protein with homology to a family of transcriptional activators [19]. PA subunit can also be translocated into the nuclei and regulate cRNA/vRNA synthesis [36]. Here, for the first time we demonstrated that PA subunit bind with PDCD7 in the nuclei and activated apoptosis pathway and induced cell apoptosis. The way how PA subunit bind with PDCD7 still needs to be further investigated.

PDCD7, also named as apoptosis-related protein ES 18, locates in chromosome 15 and encodes a 59 kDa protein that interacts with the U11 small nuclear ribonucleoprotein, a component of the minor U12-type spliceosome, and contributes to the pre-mRNA splicing of U12-type introns [37–39]. Mouse PDCD7 was first identified using the screening approach in 1999 and characterized as an apoptosis-related protein that involves in apoptotic cell death of T-cells [40]. However, the specific function of PDCD7 in human is still unknown, although several studies have reported that human PDCD7 may related to the acute myeloid leukemia (AML) [41, 42]. Compared with the non-malignant samples, higher PDCD7 expression was found in AML patients and related to lower complete remission rate, shorter relapse-free survival, and overall survival, suggesting that overexpression of PDCD7 may serve as a molecular marker for prognosis estimation of AML [41, 42]. By MS combining with Co-IP experiments, we found PDCD7 is a binding partner of PA subunit of avian H9N2 virus and mediates cell apoptosis after PA overexpression in A549 lung cells, indicating that the interaction between PDCD7 and PA subunit may contribute to the cell apoptosis during virus infection.

## Conclusion

Collectively, the PA subunit of H9N2 virus might induce lung cell death through binding and activating PDCD7, which provide new insights in the role of PA subunit during H9N2 influenza virus infection and new treatment possibilities for H9N2 infected cases. Therefore, further study should be warranted.

## Supplementary Information

**Additional file 1. Figure 1:** H9N2 infection leads to apoptosis in A549 cells. A. Flow cytometry analysis of cell apoptosis by Annexin V and PI staining in A549 cells infected with control or H9N2 virus. B. Quantitative results of cells at different stages. Early apoptosis: E-apoptosis; Late apoptosis: L-apoptosis.

## Data Availability

The data that support the findings of this study are available from the authors on request.

## Abbreviations

PDCD7: Programmed cell death protein 7
RNPs: Ribonucleoproteins
PB1: Polymerase basic 1
ATCC: American Type Culture Collection
FBS: Fetal Bovine Serum
HRP: Horseradish Peroxidase
MS: Mass spectrometry
AML: Acute myeloid leukemia
