# Supplementary figures and images for: Polymerase acidic subunit of H9N2 polymerase complex induces cell apoptosis by binding to PDCD 7 in A549 cells
Source: Virol J. 2021 Apr 13;18:75. doi: 10.1186/s12985-021-01547-7 (PMC8045253; doi:10.1186/s12985-021-01547-7)

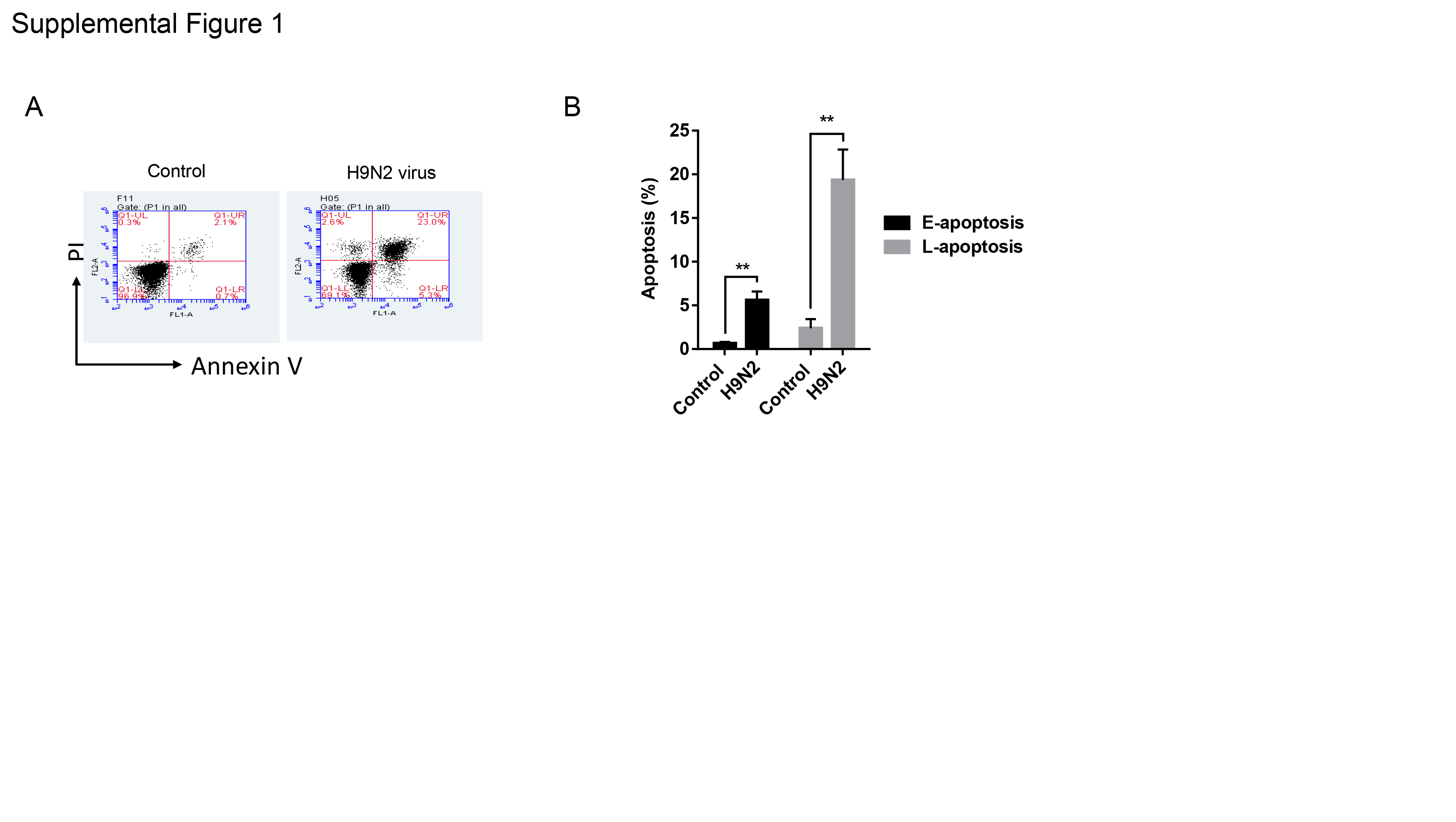

Supplement: Supplementary file 1 — Additional file 1. Figure 1: H9N2 infection leads to apoptosis in A549 cells. A. Flow cytometry analysis of cell apoptosis by Annexin V and PI staining in A549 cells infected with control or H9N2 virus. B. Quantitative results of cells at different stages. Early apoptosis: E-apoptosis; Late apoptosis: L-apoptosis. [file 12985_2021_1547_MOESM1_ESM.tif]
